# Supplementary material for: A novel approach to graph distinction through GENEOs and permutants
Source: Sci Rep. 2025 Feb 20;15:6259. doi: 10.1038/s41598-025-90152-7 (PMC11842813; doi:10.1038/s41598-025-90152-7)
Supplement: Supplementary file 1 — Supplementary Information. [file 41598_2025_90152_MOESM1_ESM.pdf]

A novel approach to graph distinction through GENEOs and permutants

Supplementary Information

Giovanni Bocchi[1,*], Massimo Ferri[2], Patrizio Frosini[3]

[1] *Department of Environmental Science and Policy*
University of Milan
Milan, MI 20133.

[2] *Department of Mathematics and ARCES*
University of Bologna
Bologna, BO 40126

[3] *Department of Computer Science*
University of Pisa
Pisa, PI 56127

[*] Corresponding author: giovanni.bocchi1@unimi.it

Supplementary Table 1: The average execution times and corresponding accuracies for $r = 4$ are presented below. Bold values indicate the most accurate method(s) in terms of accuracy. For every value of r, a maximum execution time was predetermined to prevent overly lengthy computations from occurring. Specifically, the timeout was set at 10 seconds for $r = 3$, 30 seconds for $r = 4$, and 90 seconds for $r = 5$. It is important to note that the accuracy values are influenced by this time restriction. If a method fails to conclude within the designated time frame, its result is automatically set to null, which counts as an incorrect classification of a non-isomorphic pair. As a result, methods like NTX-IS, which is an exact algorithm, may exhibit accuracy scores below 1, anyway such values are also marked bold to highlight its exactness.

Method	N									
	100		500		1000		5000		10000	
	Time	Accuracy	Time	Accuracy	Time	Accuracy	Time	Accuracy	Time	Accuracy
GENEO-1	0.041	0.994	0.214	0.996	0.441	0.994	2.172	0.998	4.374	0.998
GENEO-2	0.052	**1.000**	0.274	0.998	0.557	**1.000**	2.755	**1.000**	5.588	**1.000**
GENEO-3	0.204	**1.000**	1.096	**1.000**	2.263	**1.000**	11.369	**1.000**	22.83	**1.000**
NTX-COULD	0.004	0.926	0.042	0.878	0.149	0.882	3.299	0.858	12.948	0.882
NTX-FAST	0.002	0.924	0.007	0.878	0.014	0.882	0.074	0.858	0.149	0.882
NTX-FASTER	0.000	0.000	0.000	0.000	0.000	0.000	0.002	0.000	0.003	0.000
NTX-IS	10.188	**0.818**	30.000	**0.000**	30.000	**0.000**	30.000	**0.000**	30.000	**0.000**
1-WL	0.001	0.000	0.005	0.000	0.010	0.000	0.049	0.000	0.100	0.000
2-WL	7.123	0.000	30.000	0.000	30.000	0.000	30.000	0.000	30.000	0.000
3-WL	30.000	0.000	30.000	0.000	30.000	0.000	30.000	0.000	30.000	0.000

Supplementary Table 2: The average execution times and corresponding accuracies for $r = 5$ are presented below. Bold values indicate the most accurate method(s) in terms of accuracy. For every value of r, a maximum execution time was predetermined to prevent overly lengthy computations from occurring. Specifically, the timeout was set at 10 seconds for $r = 3$, 30 seconds for $r = 4$, and 90 seconds for $r = 5$. It is important to note that the accuracy values are influenced by this time restriction. If a method fails to conclude within the designated time frame, its result is automatically set to null, which counts as an incorrect classification of a non-isomorphic pair. As a result, methods like NTX-IS, which is an exact algorithm, may exhibit accuracy scores below 1, anyway such values are also marked bold to highlight its exactness.

Method	N									
	100		500		1000		5000		10000	
	Time	Accuracy	Time	Accuracy	Time	Accuracy	Time	Accuracy	Time	Accuracy
GENEO-1	0.102	**1.000**	0.553	**1.000**	1.120	**1.000**	5.732	**1.000**	11.378	**1.000**
GENEO-2	0.124	**1.000**	0.662	**1.000**	1.339	**1.000**	6.848	**1.000**	13.582	**1.000**
GENEO-3	0.637	**1.000**	3.578	**1.000**	7.412	**1.000**	37.224	**1.000**	74.603	**1.000**
NTX-COULD	0.005	0.994	0.054	0.964	0.189	0.936	4.133	0.934	16.087	0.934
NTX-FAST	0.002	0.990	0.010	0.962	0.019	0.936	0.101	0.934	0.216	0.934
NTX-FASTER	0.000	0.000	0.000	0.000	0.000	0.000	0.002	0.000	0.003	0.000
NTX-IS	10.623	**0.996**	89.865	**0.002**	90.000	**0.000**	90.000	**0.000**	90.000	**0.000**
1-WL	0.001	0.000	0.005	0.000	0.010	0.000	0.052	0.000	0.105	0.000
2-WL	7.119	0.000	90.000	0.000	90.000	0.000	90.000	0.000	90.000	0.000
3-WL	90.000	0.000	90.000	0.000	90.000	0.000	90.000	0.000	90.000	0.000
